# Adapting in-cell ELISA to investigate the localization of hepatitis C virus envelope glycoproteins

**DOI:** 10.1128/spectrum.02895-25

**Published:** 2026-08-11

**Authors:** Stavros Dimitriadis, Matthew Edmans, Nicole Frumento, Ng Hong Xiang, Justin Bailey, Andrea Cox, Paul Klenerman, Eleanor Barnes, Gerardo Montalvo Zurbia-Flores

**Affiliations:** 1 Oxford University Hospitals NHS Foundation Trust 6397, Oxford, United Kingdom; 2 Peter Medawar Building for Pathogen Research, University of Oxford 6396, Oxford, United Kingdom; 3 Department of Medicine, John Hopkins University, Baltimore, Maryland, USA; Barnard College, Columbia University, New York, New York, USA

**Keywords:** E1E2, vaccine, ELISA, HCV

## Abstract

The development of hepatitis C virus (HCV) vaccines remains challenging due to extensive viral genetic diversity and the complex biology of the envelope glycoproteins E1 and E2, which are major targets of broadly neutralizing antibodies and are associated with spontaneous viral clearance. Assessment of E1E2 expression is complicated by reported intracellular retention, limiting the ability to evaluate antigen localization efficiently. Here, we adapted an in-cell enzyme-linked immunosorbent assay (ELISA) to quantify membrane-associated and intracellular E1E2 expression and explored its utility as an early screening tool for HCV vaccine antigens. HEK293T and Huh-7 cells were transfected with mRNA encoding untruncated E1E2 sequences from two antigenically distinct HCV isolates, C110D0 and C176D0. Cells were analyzed following fixation alone to detect membrane-associated antigen or following fixation and permeabilization to detect total cellular antigen. In-cell ELISA reproducibly detected E1 and E2 above mock-transfected controls under both conditions. For C110D0, E1 and E2 signals were significantly greater following permeabilization than fixation alone, consistent with partial intracellular retention. Similar trends were observed for C176D0, although differences between conditions were less pronounced. Flow cytometry using anti-E2 monoclonal antibodies confirmed surface expression of E2 on a subset of transfected cells and corroborated the localization patterns by in-cell ELISA. These findings establish in-cell ELISA as a scalable and reproducible approach for evaluating the expression and localization of HCV envelope glycoproteins, and support its use in the prioritization of candidate antigens for downstream studies.

**IMPORTANCE:**

Hepatitis C virus remains a major cause of chronic liver disease and liver cancer worldwide. Although highly effective direct-acting antivirals can cure infection, access to treatment remains limited in low- and middle-income countries, with new infections continuing to occur globally. An effective vaccine is therefore likely to be critical for long-term hepatitis C virus (HCV) control. One challenge in vaccine development is determining whether viral proteins are displayed on the surface of cells, where they can be recognized by the immune system or remain hidden inside infected cells. We describe a simple laboratory assay that distinguishes between these two patterns of protein expression using standard equipment and scalable workflow. Applying this approach, we demonstrate surface expression of envelope proteins from two HCV isolates while also identifying differences in their intracellular retention. This method provides a practical tool for early evaluation and prioritization of vaccine antigens before their progression to more detailed downstream studies.

## OBSERVATION

While hepatitis C virus (HCV) has been identified as a priority pathogen for vaccine research in five out of six World Health Organization regions, no vaccine is available due to the virus’ genetic diversity and high mutation rate (1, 2). Evidence of human spontaneous clearance of HCV suggests that protective immunity could theoretically be possible (2). In this subset, spontaneous clearance has been associated with the development of broadly neutralizing antibodies (bnAbs) targeting envelope glycoproteins (E1 and E2) capable of cross-genotype neutralization (3–6). The only human vaccine trial to reach phase 2, based on a viral vector expressing HCV genotype 1b non-structural proteins, elicited T-cell responses but failed to prevent chronic infection (7). This outcome may reflect limited cross-genotype coverage or the absence of neutralizing antibody induction against E1E2. Therefore, we hypothesize that a vaccine candidate capable of eliciting bnAbs to E1 and E2 remains a promising strategy.

The E1 and E2 are transmembrane glycoproteins which form a heterodimer (E1E2) essential for HCV cell entry. E1E2 contains hypervariable domains and glycan motifs critical for immune evasion (8–10). Composed of 160 (33 kDa) and 360 (70 kDa) amino acids, both E1 and E2 possess endoplasmic reticulum (ER) retention signals, raising uncertainty whether untruncated, stable E1E2 can co-exist at the cell membrane in the absence of other viral proteins (11–13). ER retention is therefore a major obstacle for presenting E1E2 as vaccine antigens, as limited surface display may restrict their immunogenic potential. Therefore, the production of stable, native E1E2 that can be expressed on the cell surface might enhance vaccine development.

An enzyme-linked immunosorbent assay (ELISA) that quantifies spatial E1E2 antigen expression can serve as an early screening tool to prioritize vaccine candidates prior to functional testing. This approach combines scalability, quantifiability, and cost-effectiveness, making it well suited for high-throughput evaluation. By contrast, flow cytometry and confocal microscopy require state-of-the-art equipment and are expensive and labor-intensive to perform at scale. Furthermore, biotinylation assays only capture surface proteins and cannot simultaneously measure intracellular antigen. Instead, our ELISA can be performed on a standard benchtop and, importantly, can be integrated with neutralization assays to link antigen expression with functional antibody responses.

**Fig 1:**
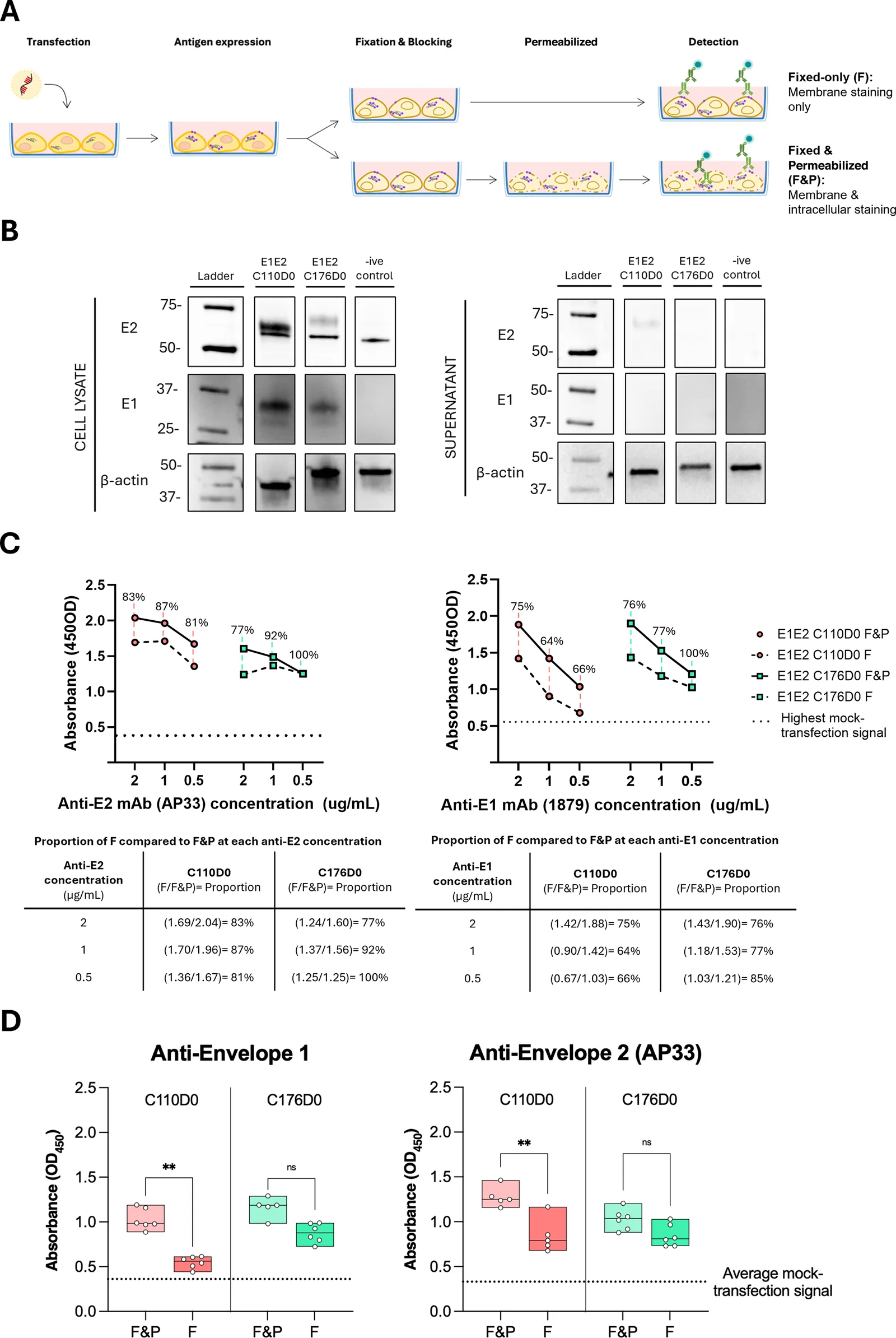
Quantification of the expression of envelope proteins from two HCV strains (C110D0 and C176D0) using in-cell ELISA. (**A**) Diagram highlighting the key steps of the in-cell ELISA. (**B**) Cropped immunoblots show detection of E1 and E2 in cell lysates and supernatants from HEK293 cells transfected with mRNA encoding E1E2. Detection of β-actin was used as a loading control. Immunoblots represent three technical replicates. (**C**) Optimization of mAb concentration (0.5, 1, and 2 μg/mL) to measure the expression of E1 and E2 using in-cell ELISA. Values shown represent the average signal of technical duplicates following the subtraction of the matched negative control's background signal. Solid and dashed lines represent F&P and F conditions, respectively. The dotted line represents the highest signal from the mock-transfected cells (threshold of positive detection). Dotted lines between the F and F&P conditions and associated percentages correspond with the proportion of the F to F&P signal as a percentage. The calculations are included in the tables below each graph. (**D**) Quantification of expressed E1 and E2 proteins using anti-AP33 and anti-E1 mAbs (1 μg/mL) in an in-cell ELISA panel. The assay included six technical replicates; single-well measurements are represented by dots. The median and ±interquartile range are shown. The dotted line represents the highest signal from the mock-transfected cells (threshold of positive detection).

Hence, we adapted an in-cell ELISA to investigate potential differences in the retention and localization of E1E2 antigens derived from two antigenically different HCV isolates (C110D0 and C176D0). The assay is described in detail in the Supplemental methods S1 and illustrated in Fig. 1A. Briefly, whole cells are transfected with mRNA encoding HCV’s E1E2, incubated, and treated in two conditions: in the fixed condition (F), cells are fixed with paraformaldehyde prior to staining with E1 or E2-specific antibodies, whereas in the fixed and permeabilized (F&P) condition, fixed cells are permeabilized prior to staining. Staining of fixed cells allows the quantification of antigens present only on the cell surface, whereas staining of F&P cells allows the detection of both intracellular antigens and on the cell surface.

Firstly, we obtained two unformulated mRNA vaccine candidates (mRNA Access, Moderna) encoding untruncated HCV E1E2 sequences that were transfected into HEK293T or Huh-7 cells using a lipofectamine-based protocol. Antigen expression was confirmed using immunoblotting (Fig. 1B). Transfected cells demonstrated E2 antigen expression at 70 kDa. C110D0 exhibited E1 expression at 30 and 35 kDa, while C176D0 showed expression primarily at 35 kDa. C110D0 produced more evident E1 and E2 expression compared to C176D0.

When using in-cell ELISA, E1 and E2 proteins were detected in both F and F&P conditions (Fig. 1C). C110D0-E2 and E1 were detectable at all antibody dilutions tested. E2 expression was consistently higher across all dilutions in the F&P condition, with the proportion of F to F&P ranging from 81% to 87%, suggesting successful detection of intracellular E2. A similar pattern was observed for C176D0-E2 expression, although the distinction between F&P and F conditions is less clear at lower antibody concentrations, with the 0.5 μg/mL reaching 100%. C110D0 also produced detectable E1, with consistently higher expression when F&P. Similar expression patterns were noted for C176D0-E1.

Having identified consistent antibody binding to expressed E1 and E2 using 1 μg/mL antibody concentration, we performed three experimental replicates of the in-cell ELISA, each with six technical replicates per condition to capture variation (Fig. 1D). E1 and E2 were consistently detected above the mock transfection threshold in both the F&P and F conditions. The expression of C110D0-E2, but not C176D0-E2, was found to be significantly higher in the F&P condition compared to the F-only condition. Similarly, detection of C110D0-E1 was significantly higher in the F&P condition compared to F when binding to E1 was measured.

**Fig 2:**
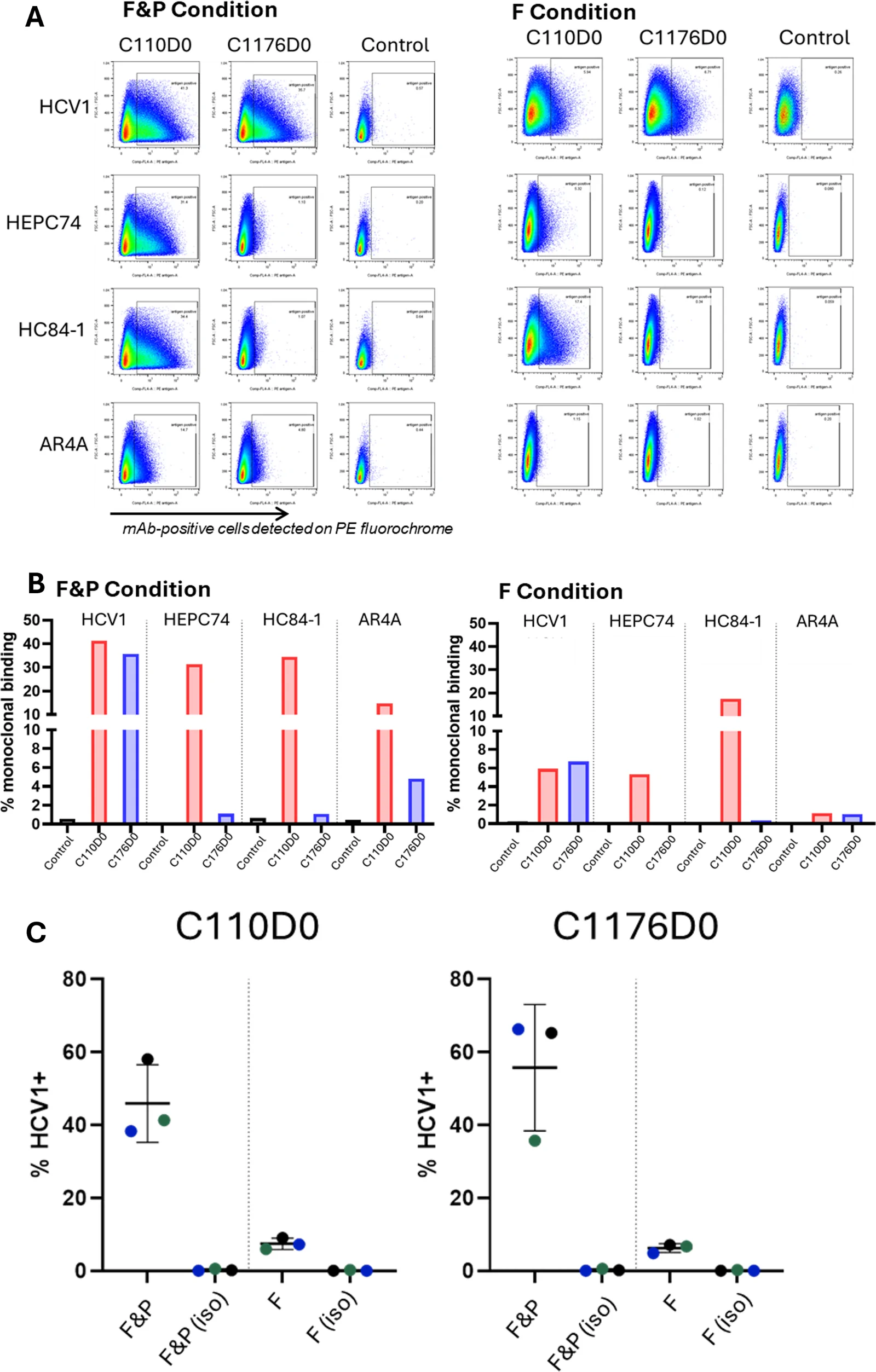
Validation of the in-cell ELISA using flow cytometry. (**A**) Detection of HCV E2 and E1E2 in C110D0- and C176D0-transfected cells using a panel of anti-E2 mAbs in flow cytometry. A mock transfection represents a negative control of protein expression. The mAb panel (HCV1, HEPC74, HC84-1, and AR4A) was tested in F&P and F conditions. (**B**) Quantification of the proportion of E2-positive cells detected by each antibody presented in panel A. Data shown are representative of three experimental replicates. (**C**) Proportion of C110D0- and C176D0-transfected cells bound by anti-HCV1 mAb. Dots represent technical replicates, grouped per condition. Mean and standard deviation within replicates are shown.

To validate the outcome of the ELISA, we used flow cytometry on transfected HEK293T cells under the same conditions (F and F&P) using multiple anti-E2 monoclonal antibodies, as well as the anti-AR4A antibody that requires folded E1E2 to bind. C110D0-E2 was detected in the F and F&P conditions by all anti-E2 antibodies (Fig. 2A and B). C176D0-E2 was detected by anti-HCV1 in the F&P and in the F conditions. C176D0-E2 is not recognized by HEPC74 or HC84-1, in keeping with internal data shared by the Bailey lab. Across all antibodies tested, anti-E2 monoclonal binding was greater in the F&P condition compared to the F condition, suggesting that many of the transfected cells only express E2 intracellularly. Anti-AR4A binding was demonstrated in both C110D0 and C176D0 in the F&P condition; however, only 1% of C110D0 and C176D0 cells were AR4A positive in the F condition. This might be due to anti-AR4A not being able to bind membrane-bound E1E2 or due to extracellular E2 not coupling with E1 to form heterodimers. Three experimental replicates using HCV1 to quantify E2-binding in flow cytometry showed consistent outcomes, confirming the reproducibility of our findings (Fig. 2C).

In summary, we established an in-cell ELISA protocol to quantify antigen expression of HCV vaccine candidates *in vitro* that can distinguish differences in the spatial localization of expressed antigens (intracellularly or membrane bound). Using technical and experimental replicates, we showed the potential of this assay for scalability while maintaining reproducibility. Compared to other techniques, in-cell ELISA can offer scalability at a cheaper cost and be combined with other assays, such as neutralization assays or monoclonal panel testing, to complement its data outputs. In this instance, C110D0 and C176D0 E1 and E2 antigens were detected on the cell membrane, suggesting that expressed E1E2 might not be exclusively retained in the ER. Our in-cell ELISA data were corroborated with flow cytometry, which showed a proportion of cells expressed E2 on the cell surface. This protocol is unique in being specifically applied to inform the selection of antigens for the development of vaccine candidates. While further work is required to investigate the impact of these findings, comparative studies of the expression and localization of E1E2 from different origins might prove informative for the selection of antigens of future vaccine candidates.

## SUPPLEMENTAL MATERIAL

Supplemental methods spectrum.02895-25-s0001.docx Expanded methodology.

ASM does not own the copyrights to Supplemental Material that may be linked to, or accessed through, an article. The authors have granted ASM a non-exclusive, world-wide license to publish the Supplemental Material files. Please contact the corresponding author directly for reuse.
