# Supplementary material for: Adapting in-cell ELISA to investigate the localization of hepatitis C virus envelope glycoproteins
Source: Microbiol Spectr. 2026 Aug 11;14(9):e02895-25. doi: 10.1128/spectrum.02895-25 (PMC13532185; doi:10.1128/spectrum.02895-25)
Supplement: Supplementary file 1 [file spectrum.02895-25-s0001.docx]

**Supplemental methods**

**Cell Culture**

HEK293T and Huh-7 cells were cultured at 37^o^C in a humidified environment at 5% CO_2_ in Dulbecco Modified Eagle Medium (DMEM), (10% Foetal Calf serum (FCS), 1% Penicillin/Streptomycin, 1% L-Glutamine).

**Immunogen design and vaccines**

We obtained two unformulated mRNA vaccines (mRNA Access, Moderna) encoding full-length (555 amino acids) HCV E1E2 sequences, approximately corresponding to 192-383 (E1) and 384-746 (E2) of two antigenically different sequences (C110D0 and C176D0) obtained from HCV-infected patients and provided by Dr. Justin Bailey (John’s Hopkins, USA).

**mRNA transfection**

440,000 cells/well were seeded in a 6-well plate and 20,000 cells/well were seeded in a 96-well plate for immunoblotting or in-cell ELISA, respectively. After overnight incubation (37^o^C, 5% CO_2_), cells were transfected accordingly: 1) For Western Blot and Flow cytometry, 1μg mRNA and 10μL of Lipofectamine-2000 were diluted in up to 100μL Opti-MEM medium per well. 2) For in-cell ELISA, 100ng mRNA and 0.5μL of Lipofectamine-2000 were diluted in up to 10μL Opti-MEM medium per well. 3) Negative controls were prepared using equivalent volumes of nuclease-free water instead of mRNA. The media was aspirated from cell culture plates and exchanged with Opti-MEM. Then, the mRNA-lipofectamine mixture was added to each well (100μL for Western Blot, 10μL for in-cell ELISA).

**Western Blot**

HEK293T cells were incubated for 24 hours following mRNA transfection as described above. During harvest, samples are collected, spun down, and separated in cells and supernatant fractions. Samples were processed under denaturing conditions (100^o^C, SDS and β-mercaptoethanol) prior to testing or storage. Samples were loaded on SDS-PAGE (Mini-Protean TGX gels, BIORAD) and subject to electrophoresis under reduced-conditions. After proteins were transferred to PVDF membranes (Trans-Blot Turbo transfer system, BIO-RAD), membranes were blocked (5% skim milk in 0.1% PBS-T) and incubated with primary and secondary antibody solutions. Membranes were developed using Clarity ECL Western Blotting Kit (BIO-RAD) prior to image capture. Western blot experiments were repeated on separate days to obtain three technical replicates.

**In-Cell ELISA**

Huh-7 cells were seeded into 96-well plates and transfected as described above. After 24 hours, the media was aspirated, and cells were fixed with 4% formaldehyde at room temperature. Cells were then washed three times (PBS). The plate was divided in two, and those cells to be permeabilised were exposed to a 1% PBS-Triton-X solution, shaking at 300rpm at room temperature. After, permeabilization, all cells were blocked (5% skim milk in 1% PBS-T) regardless of their treatment and incubated shaking at room temperature for 2 hours. Primary antibody was serially diluted in blocking buffer to achieve a range of concentrations between 0.5μg/mL and 2μg/mL. 100μL of antibody solution was used per well, except for 1 well per treatment arm that was incubated with blocking solution as a control. Cells were incubated overnight at 4^o^C and then washed 5 times with 200μL of PBS- 1%Tween. Secondary antibody was prepared in blocking solution and 100uL was used per well, including the wells without any primary antibody. Cells were incubated in secondary antibody for 2 hours at RT prior to washing 5 times with PBS-1% Tween. Plates were developed using 3,3’,5,5’-tetramethylbenzidine (TMB) and absorbance was detected at 450nm. Analysis was performed by subtracting the signal from the secondary antibody control, as well as the signal produced by lipofectamine-only cells.

After antibody concentrations had been identified, experimental replicates with six technical replicates were performed by separate researchers on separate days using cells from different passages. Statistical analysis was performed on a per-run basis as a semi-quantitative assay using non-parametric Kruskal-Wallis test.

**Flow Cytometry**

HEK-293T cells were incubated for 24 hours following mRNA transfection as described above. Following transfection, cells were harvested using PBS, centrifuged and resuspended in FACS buffer. Cells were harvested and resuspended in FACS buffer for counting. Cells were then fixed with 100μL BD Cytofix Fixation Buffer (containing paraformaldehyde). Cells in F-only condition were washed with PBS, whereas cells in the F&P condition were further permeabilised by incubating using 100μL BD Perm buffer for 10 minutes. All cells were stained with anti-E2 HCV1 antibody for 20minutes at 4^o^C. Cells were then washed with either Perm/wash buffer (F&P) or PBS (F-only) prior to incubating with PE anti-human IgG for 30 minutes. Cells were washed twice with either Perm/wash buffer (F&P) or PBS (F-only) and resuspended in FACS buffer. Samples were run using the MACSquantX. Data was analysed using Flowjo v10. Three experimental replicates were performed of the flow cytometry data.

**Antibodies**

| **Antigen** | **Species** | **Clone** | **Labelling Strategy** | **Source** | **Staining concentration** |
| --- | --- | --- | --- | --- | --- |
| E2 | Goat | Polyclonal | WB  Primary antibody | Abcam | 1:5000 |
|  | Mouse | AP33 | In-Cell ELISA  Primary antibody | Merck/Sigma | Serial dilution |
|  | Human | HCV-1 | Flow Cytometry  Primary antibody | Provided by Bailey lab and produced in-house | 1ug/mL |
|  | Human | HEPC74 | Flow Cytometry  Primary antibody | Provided by Bailey lab and produced in-house | 1ug/mL |
|  | Human | HC84-1 | Flow Cytometry  Primary antibody | Provided by Bailey lab and produced in-house | 1ug/mL |
| E1E2 | Human | AR4A | Flow Cytometry  Primary antibody | Provided by Bailey lab and produced in-house | 1ug/mL |
| E1 | Mouse | 1879 | WB and In-Cell ELISA Primary antibody | Native Antigen | WB 1:1000  Serial dilution for In-Cell |
| Β-actin | Mouse | AC-15 | WB Primary antibody (Loading control) | Merck/Sigma | 1:50000 |
| Mouse IgG-HRP | Goat | Polyclonal | Secondary antibody | Bio-Rad | WB 1:5000  In-Cell 1:2000 |
| Anti-Goat IgG- HRP | Rabbit | Polyclonal | Secondary antibody | Abcam | WB 1:5000 |
| Anti-human IgG-PE | Goat | Polyclonal | Flow Cytometry Secondary antibody | ThermoFisher | 1ug/mL |

**Data Availability**

HCV E1E2 sequences are available in GenBank (accession numbers C110D0-MH834920.1 and C176D0-KX621468.1). All other data are available from the corresponding author upon reasonable request. Vaccine materials generated by Moderna are subject to MTA restrictions.
